# New paradigm for aging research: aging studies through innovative AI applications and interdisciplinary collaborations

**DOI:** 10.1093/lifemedi/lnaf039

**Published:** 2026-02-19

**Authors:** Zeyu Gao, Mei Li, Jingyi Li, Moshi Song

**Affiliations:** State Key Laboratory of Organ Regeneration and Reconstruction, Institute of Zoology, Chinese Academy of Sciences, Beijing 100101, China; Beijing Institute for Stem Cell and Regenerative Medicine, Beijing 100101, China; State Key Laboratory of Organ Regeneration and Reconstruction, Institute of Zoology, Chinese Academy of Sciences, Beijing 100101, China; Beijing Institute for Stem Cell and Regenerative Medicine, Beijing 100101, China; University of Chinese Academy of Sciences, Beijing 100049, China; State Key Laboratory of Organ Regeneration and Reconstruction, Institute of Zoology, Chinese Academy of Sciences, Beijing 100101, China; Beijing Institute for Stem Cell and Regenerative Medicine, Beijing 100101, China; University of Chinese Academy of Sciences, Beijing 100049, China; State Key Laboratory of Organ Regeneration and Reconstruction, Institute of Zoology, Chinese Academy of Sciences, Beijing 100101, China; Beijing Institute for Stem Cell and Regenerative Medicine, Beijing 100101, China; University of Chinese Academy of Sciences, Beijing 100049, China

The global population is aging at an accelerating rate, presenting a critical public health challenge worldwide. At the 2025 China Aging Science Conference and International Conference on Aging Biology, which brought together over 1200 participants, leading international experts convened to examine the multifaceted obstacles confronting aging research. A significant research gap was identified, as current approaches remain fragmented and lack integrated frameworks capable of synthesizing complex multi-scale data and effectively translating discoveries into clinical practice. A consensus emerged around the imperative to move beyond disciplinary silos and harness advanced artificial intelligence (AI) to drive interdisciplinary collaboration.

Accordingly, an AI-enhanced, cross-sector collaboration paradigm was proposed to address this gap by unifying heterogeneous data streams, bridging translational divides, and accelerating the development of actionable interventions for aging. Experts highlighted persistent challenges in integrating diverse datasets and translating laboratory breakthroughs into clinical practice, calling for the establishment of platforms that bridge academia, clinical medicine, and industry. The conference concluded with a shared commitment to developing clear, actionable pathways to transform scientific discoveries into real-world applications and market-ready solutions, ultimately aiming to promote healthy aging and address age-related diseases through coordinated global efforts.

## Innovative applications and prospects of AI in aging research

Aging research has increasingly become a priority in response to the escalating global demographic shift toward older populations. Characterized by progressive functional decline across organs and tissues, aging is the primary risk factor for chronic diseases. The accelerating prevalence of age-related conditions strains healthcare systems and economic development. Concurrently, rapid advances in AI are transforming the field. By integrating multi-omic data, identifying aging biomarkers, and modeling molecular interactions, AI accelerates the elucidation of aging mechanisms, expedites aging intervention strategy development, and refines therapeutic protocols, thus reshaping aging research paradigms.

Dr. Gang Pei opened the discussion by proposing an expansion of “large models” beyond AI to encompass social ecosystems. He argued that such models should incorporate economic structures and intergenerational dynamics, noting that generational bonds form a critical “wheel of willingness.” While acknowledging AI’s value as a research tool, Dr. Pei maintained that human researchers must remain central in leading the substantive exploration of aging’s complexities.

Dr. Songling Wang proposed a developmental biology lens for aging prevention, emphasizing AI’s role in analyzing developmental and aging processes. He also underscored the importance of homeostatic medicine, exemplified by nitrate’s regulatory function in maintaining cellular balance, and called for AI-aided research to uncover the causes, mechanisms, and solutions for age-related homeostasis imbalance.

Dr. Jing-Dong Jackie Han then highlighted AI’s dual applications in population-scale and single-cell aging studies. While acknowledging persistent challenges in population data alignment and cleaning, she projected confidence that expanding datasets would mitigate these limitations, reinforcing AI’s scalability in aging research.

Dr. Riqiang Yan exemplified AI’s disruptive potential through Alzheimer’s β-secretase inhibitor discovery, contrasting traditional methods’ inefficiency with AI-driven small-molecule design as the field’s imperative direction.

Dr. Danica Chen expanded on this, detailing AI’s multidimensional impact in translating basic research. She highlighted its “extensive prospects,” spanning from biological age quantification and biomarker discovery to drug repurposing and molecule design.

Confronted with the vast volume and complexity of aging and neurological disease data, Dr. Xu Zhang emphasized AI’s indispensability for distilling key biological signals and testing mechanistic hypotheses. To address this, he called for the creation of a dedicated “aging big model” specifically designed for the field.

Dr. Eric Gilson emphasized the inherent variability of aging trajectories shaped by species divergence and socioeconomic factors. To decode this complexity, he proposed the use of large-scale longitudinal cohorts analyzed through AI-enabled integrative frameworks, positioning AI as essential for modeling heterogeneous aging processes and systematically discovering biomarkers.

Dr. Yong Shen mapped AI’s dual challenges: parsing multi-organ/multi-omic nonlinearity while overcoming heterogeneity via cross-racial and cross-environmental models to extract universal principles. He envisioned organ-specific biomarker warning systems to bridge aging discovery and intervention, stressing that AI must overcome data overload by synthesizing layered biological and clinical information to decode aging’s underlying network logic.

Dr. Zhongjun Zhou highlighted that limited aging data, with sparse dynamic datasets and unclear causal validation, sometimes hampers our mechanistic understanding of aging. He proposed a shift to synthetic biology’s systems approach, integrating AI with intervention-driven models (e.g., targeted pathway perturbation). This approach, he argued, would advance the field beyond fragmented observational methods, evolving toward a unified framework for aging complexity.

Overall, the experts collectively underscored AI’s transformative potential in aging research through applications like biomarker discovery, drug development, and complex data analysis. However, significant challenges were also highlighted, including data limitations, complexity in modeling multi-system interactions, and data overload. To fully harness AI’s power, they advocated for developing tailored AI tools for aging research, adopting integrative and systems approaches, building large longitudinal cohorts, and crucially, maintaining human leadership in guiding research, so as to moving aging research toward more unified and scalable frameworks.

## Promoting interdisciplinary synergy to advance aging research

As a complex process of multisystem functional decline, aging underpins the majority of chronic diseases and imposes escalating burdens on healthcare infrastructure and societal resources. Confronting these challenges necessitates transcending traditional siloed methodologies. Consequently, the integration of disciplines spanning biology, medicine, data science, engineering, social sciences, and policy presents an unparalleled opportunity for transformative breakthroughs.

Building on the imperative for cross-disciplinary integration, Dr. Gang Pei posited that aging’s complexity constitutes both a challenge and catalyst for research paradigm shifts. He championed regenerative models acknowledging aging’s inherent uncertainty and diversity, while emphasizing that multidisciplinary collaboration is essential to address this paramount scientific problem.

Dr. Jing-Dong Jackie Han envisioned AI-driven facial recognition as a tool to assess organ-specific aging states, noting that facial features reflect systemic decline. While AI can mine biomarkers from population data, she pointed out that effective interventions require multidimensional strategies, including lifestyle modifications, small-molecule therapies, and synthetic biology, underscoring aging research’s fundamental reliance on interdisciplinary collaboration.

Dr. Danica Chen prioritized dismantling interdisciplinary communication barriers through cross-domain workshops and training programs. She also endorsed policies that foster collaboration, such as dedicated funding for team-based problem-solving initiatives.

Dr. Eric Gilson highlighted the importance of studying aging across diverse species, including animals, plants, and bacteria, to uncover longevity mechanisms and biomarkers. He further proposed extending aging research to ecosystems by examining interspecies interactions and ecological imbalances, such as the health impacts of COVID-19.

Dr. Yong Shen used Alzheimer’s research to illustrate the importance of shifting from a brain-centric pathology focus to multi-organ studies, such as examining the brain–gut–liver–bone axes. He noted that significant progress has been made in the field of neurodegenerative disease research, which clearly illustrates the immense value of integrating interdisciplinary perspectives.

Dr. Zhongjun Zhou stressed that interdisciplinary success hinges on paradigm shifts in scientific thinking, urging rigorous integration of various disciplines to decode aging mechanisms. Highlighting the persistent gap between basic and clinical research, he also emphasized the need for clinically driven questions to bridge translational divides and ensure tangible societal impact.

In summary, the experts jointly emphasized the significance of interdisciplinary cooperation in aging research and proposed a variety of strategies, such as breaking down communication barriers through cross-disciplinary workshops and training programs, using AI to assess aging states, studying aging mechanisms across different species and ecosystems, and exploring diseases from a multi-organ perspective. These perspectives collectively highlight that interdisciplinary collaboration is essential for achieving breakthroughs in aging research, generating tangible social impacts, and alleviating the burdens of age-related diseases.

## Translating aging research from bench to bedside

Translating aging research into clinical practice is crucial for addressing the health challenges posed by an aging population. While we have made progress in understanding aging, turning this knowledge into practical solutions remains complex. Effective translation requires collaboration across the many disciplines of science, medicine, policy, industry, and others. This can help bridge the gap between research findings and real-world applications, ultimately improving health outcomes and reducing the burden of age-related diseases.

Dr. Riqiang Yan emphasized that translating basic aging research into clinical practice requires integrating mechanistic studies with thorough clinical data analysis. He stressed that scientific perseverance and rigorous analysis are essential to advance from theory to practice, even when initial findings do not align with expectations.

Dr. Xu Zhang identified breaking down translational barriers between universities, research institutes, hospitals, and enterprises as fundamental to biomedical innovation. By integrating clinical resources with basic research, they have established a research-oriented hospital system that actively engages clinical scientists, ensures systematic collection of human samples and case data, and delivers a core advantage irreplaceable by animal models.

Dr. Songling Wang emphasized the critical role of well-designed policy breakthroughs in bridging the gap between laboratory achievements and industrial investment. He called for a collaborative effort among the government, industry associations, and enterprises to create a transformational ecosystem. Through policy innovation and cooperative platforms, he called for the dismantling of barriers and the conversion of scientific research into both industrial and societal benefits.

In brief, the experts highlighted the importance of translating aging research into clinical practice through interdisciplinary collaboration and policy support. They emphasized the need to bridge the gap between aging-related laboratory research and real-world applications by integrating basic science with clinical resources and leveraging policy innovation to create an ecosystem that accelerates aging-related biomedical advances and delivers societal benefits for aging populations.

## Summary

The panel concluded that advancing aging research requires harnessing AI and deepening interdisciplinary collaboration. Experts emphasized integrating AI with multi-omics data, fostering cross-disciplinary synergy to tackle aging’s complexities, and adopting translational strategies to connect research with practical applications (Table 1). However, significant challenges persist: AI is constrained by scarce longitudinal data, difficulties in modeling nonlinear multi-system interactions, and limited interpretability; interdisciplinary collaboration is hindered by divergent scientific cultures, inequitable credit-sharing models, and insufficient funding for high-risk convergent work; and clinical translation faces obstacles in biomarker validation, regulatory navigation, and sustainable commercialization.

**Table 1.** Key insights from the panel discussion on aging research

Topics	Highlights
**Innovative applications and prospects of AI in aging research**	**Multidimensional mechanism decoding**: AI accelerates the decoding of aging mechanisms by integrating multi-omics data.
	**Expedited drug design**: AI transforms drug discovery and repurposing, significantly shortening research and development cycles.
	**Revolutionizing intervention strategies**: AI enables biological age quantification and early-warning systems, facilitating precision interventions.
**Promoting interdisciplinary synergy to advance aging research**	**Breaking disciplinary barriers**: Promote collaboration through cross-disciplinary platforms and policy-supported funding.
	**Multi-species/organ studies**: Achieve a holistic understanding of aging by leveraging multi-species and multi-organ systems.
	**Social science integration**: Incorporate social sciences into aging models to enrich research perspectives.
**Translating aging research from bench to bedside**	**Building translational ecosystems**: Establish connections between academia, hospitals, and industry for aging research.
	**Policy innovation:** Create policy breakthrough to break down barriers and accelerate translating aging research into practice.
	**Rigorous clinical-oriented science:** Integrate mechanistic studies with clinical data analysis to address clinically driven aging-related questions.

To address these barriers, the panel outlined an integrated path forward focused on two priority directions: first, building open, standardized, and shared data infrastructures to support high-quality, AI-ready datasets across the field; and second, creating catalytic programs that combine cross-disciplinary training, dedicated funding mechanisms, and public–private partnerships to systematically lower collaboration barriers and accelerate the translation of discoveries into interventions. Through such coordinated efforts, the panel envisions a collaborative ecosystem capable of transforming aging research into tangible advances in healthspan and societal well-being.

